# The TAM receptor tyrosine kinases Axl and Mer drive the maintenance of highly phagocytic macrophages

**DOI:** 10.3389/fimmu.2022.960401

**Published:** 2022-07-29

**Authors:** Lidia Jiménez-García, Christopher Mayer, Patrick G. Burrola, Youtong Huang, Maxim N. Shokhirev, Greg Lemke

**Affiliations:** ^1^ Molecular Neurobiology Laboratory, The Salk Institute for Biological Studies, La Jolla, CA, United States; ^2^ Razavi Newman Integrative Genomics and Bioinformatics Core, The Salk Institute for Biological Studies, La Jolla, CA, United States; ^3^ Molecular Neurobiology Laboratory, Immunobiology and Microbial Pathogenesis Laboratory, The Salk Institute for Biological Studies, La Jolla, CA, United States

**Keywords:** apoptotic cells, thymocyte selection, phagocytosis, iron recycling, erythropoiesis, thymus

## Abstract

Many apoptotic thymocytes are generated during the course of T cell selection in the thymus, yet the machinery through which these dead cells are recognized and phagocytically cleared is incompletely understood. We found that the TAM receptor tyrosine kinases Axl and Mer, which are co-expressed by a specialized set of phagocytic thymic macrophages, are essential components of this machinery. Mutant mice lacking Axl and Mer exhibited a marked accumulation of apoptotic cells during the time that autoreactive and nonreactive thymocytes normally die. Unexpectedly, these double mutants also displayed a profound deficit in the total number of highly phagocytic macrophages in the thymus, and concomitantly exhibited diminished expression of TIM-4, CD163, and other non-TAM phagocytic engulfment systems in the macrophages that remained. Importantly, these previously unrecognized deficits were not confined to the thymus, as they were also evident in the spleen and bone marrow. They had pleiotropic consequences for the double mutants, also previously unrecognized, which included dysregulation of hemoglobin turnover and iron metabolism leading to anemia.

## Introduction

The TAM receptor tyrosine kinases (RTKs) Axl and Mer (gene name *Mertk*) play two key roles in macrophages and other immune sentinels (1, 2). First, in concert with their ligands Gas6 and Protein S (Pros1) (3), they mediate the recognition and phagocytic engulfment of apoptotic cells (ACs) (4–6). Mer, which is expressed by all phagocytic macrophages, has repeatedly been shown to be especially important for this process (4, 5). Second, they and their ligands act as cell-intrinsic negative feedback inhibitors to suppress Toll-like and cytokine receptor signaling in dendritic cells and macrophages at the termination of the innate immune response (1, 2, 7). Both of these functions are presumably critical to the postnatal development of the thymus, since this primary lymphoid organ is the site of massive cell death during the extended period of T cell selection (8, 9). Large numbers of developing thymocytes are deleted during this developmental window, either by negative selection because they are autoreactive (exhibit hyperactive T cell receptor signaling) or by neglect because they are non-responsive (exhibit hypo- or inactive signaling) (10–12). While Mer signaling has been implicated in thymic selection of autoreactive T cells in non-obese diabetic mice (13), TAM receptor functions in the normal postnatal thymus have not to date been assessed experimentally.

We used immunohistochemistry and flow cytometry to localize Axl and Mer to several distinct immune sentinels in the thymus, and identified a prominent population of F4/80^+^CD11b^lo^ macrophages, abundant in the thymic cortex, that strongly express both of these receptors. Earlier studies have shown that this thymic macrophage population is highly phagocytic (14). In keeping with their established functions in inflammatory regulation and phagocytosis in other tissues (1, 5, 7), concerted genetic inactivation of both the *Axl* and *Mertk* genes in the mouse led to the pronounced elevation of thymic interleukin IL-1β, IL-12p40, interferon γ, and other inflammatory cytokines, and to an exuberant accumulation of ACs in the thymic cortex. In addition, RNA-seq analyses detected a marked reduction in the expression of macrophage core signature genes in the *Axl^-/-^Mertk^-/-^* thymus, a reduction that we found to be caused by a previously unrecognized deficit in the number of highly phagocytic tissue-resident macrophages (TRMs) themselves. This was in turn coupled to reduced expression of multiple phagocytosis mediators in the *Axl^-/-^Mertk^-/-^* macrophages that remained.

Importantly, we found that these pleiotropic macrophage deficits were not specific to the thymus but extended to other tissues in the body, including the bone marrow and spleen. The most prominent Axl^+^Mer^+^F4/80^+^CD11b^lo^ cells in the latter tissue are red pulp macrophages (RPMs) (15, 16), among whose principal functions are the phagocytosis of damaged and senescent erythrocytes, which are loaded with hemoglobin and iron (17–19). We found that the storage of ferritin-bound ferric (Fe^3+^) iron, which is normally prominent in RPMs, was undetectable in the *Axl^-/-^Mertk^-/-^* spleen, and was instead shunted to the epithelial cells of the proximal tubules of the kidney. This perturbation had multiple follow-on consequences, including the development of overt anemia and apparently diminished erythropoiesis. Together, these unexpected results demonstrate that concerted Axl and Mer signaling is required for the differentiation and maintenance of constitutively active, highly phagocytic TRMs throughout the body.

## Materials and methods

### Mice

All animal procedures were conducted according to guidelines established by the Salk Institute Animal Care and Use Committee. Mice were bred and housed in the Salk Institute Animal Facility under a 12-hr light/dark cycle and given ad libitum access to standard rodent chow and water. C57BL6/J mice were obtained from The Jackson Laboratory. The *Axl^-/-^*, *Mertk^-/-^*, *Axl^-/-^Mertk^-/-^*, *Axl^-/-^Tyro3^-/-^*, *Tyro3^-/-^ Mertk^-/-^*, *Tyro3^-/-^Axl^-/-^Mertk^-/-^*, and *Gas6^-/-^* mice were described previously (2, 20, 21). All lines have been backcrossed for >10 generations on to a C57BL/6 background. 4–5-week-old females (1 month old, 1 mo) were used unless figure legends indicate the use of older mice. In that case, males and females were randomly allocated to experimental groups. Wild-type and mutant mouse lines were housed separately. In general we collected a single set of tissues from individual mice for the experiments and analyses of interest.

### Immunoblotting

Tissues were snap frozen in liquid nitrogen. Frozen tissues were lysed in RIPA buffer with Halt protease and phosphatase inhibitor cocktail (Thermo Scientific) for 30min on ice. After samples were spun down at 12,000 rpm for 5min, supernatants were stored at -80°C. For immunoblots, equal amounts of protein (20 μg) in 3xLaemmli sample buffer with 0.1M DTT were subjected to electrophoresis on 4–12% Bis-Tris polyacrylamide gels (Novex, Life Technologies) and transferred to polyvinylidene fluoride (PVDF) membranes (Millipore). Membranes were blocked using 1% casein in PBS (Bio Rad) for 1h at 22-24°C and immunoblotted overnight at 4°C with primary antibodies diluted 1,000-fold in blocking buffer. Blots were then washed in TBST (50 mM Tris-HCl pH 7.5, 0.15 M NaCl, and 0.25% Tween-20) and incubated for 1 h at 22-24°C with secondary HRP-conjugated antibodies diluted 10,000-fold in blocking buffer. After washing the membrane, signal was detected with a luminol-based enhanced chemiluminescence substrate for detecting HRP (SuperSignal West Pico PLUS substrate, Thermo Scientific.). The antibodies used in immunoblotting are shown in **Supplemental Table 1**.

### Immunohistochemistry and histochemistry

For immunohistochemistry, tissues were fresh frozen in OCT tissue freezing medium and cut into 11 μm sections, air-dried and stored desiccated at -80°C. Prior to staining, sections were fixed for 4 min with ice-cold acetone, washed in PBS 0.1% Tween-20 and non-specific bindings were blocked by 1h incubation in blocking buffer (PBS containing 0.1% Tween-20, 5% donkey serum and 2% IgG-free BSA). Slides were incubated overnight at 4°C with primary antibody diluted in blocking buffer, then washed in PBS 0.1% Tween-20 and incubated with fluorophore-coupled secondary antibodies diluted in blocking buffer for 1h at 22-24°C in dark. Subsequently, slices were incubated 5 min with Hoechst diluted 1:1000 in PBS 0.1% Tween-20, then washed, sealed with Fluoromount-G (SouthernBiotech) and stored at 4°C. Images were taken on a Zeiss LSM 710 microscope with Plan-Apochromat 20x/0.8 M27 objective. The antibodies used in immunohistochemistry are shown in **Supplemental Table 1**.

For Hematoxylin-Eosin and Prussian Blue staining, tissues were fixed frozen after routine perfusion procedures. For all tissue analyses, mice were anesthetized with a final concentration of 100mg/kg Ketamine/10mg/kg Xylazine in accordance with IACUC guidelines. Mice were then transcardially perfused with 20U/ml heparin in PBS followed by freshly prepared 4%PFA in PBS. Tissues were post-fixed in 4% PFA in PBS overnight at 4°C and subsequently infiltrated in 30% sucrose in PBS for 1 day at 4°C and flash frozen in TBS tissue freezing medium. Tissues were then cut into 11 μm sections, air-dried overnight and processed for staining. For Prussian blue iron staining, slides were incubated for 5 min in 10% Potassium Ferrocyanide and then 30 min in equal parts of a freshly prepared solution containing 10% Potassium Ferrocyanide and 20% HCL. Slides were then washed and counterstained with neutral red. Finally, slides were dehydrated and mounted in VectaMount (Vector). Images were taken on an Olympus BX40 microscope, with 4x and 20x objectives.

### Imaging data analysis

For cleaved caspase 3 (cCasp3)-positive area and ratio of cortex/medulla quantification, three sections per thymus 100μm apart were analyzed with ImageJ software (version: 2.1.0). To determine cCasp3 positive area, three images of the cortex and three of the medulla were taken per section. Analyze particle tool was used to calculate the percentage of positive area per field. To determine the ratio cortex/medulla, two images that covered the entire thymic section were analyzed. The areas of interest (cortex, medulla and total area) were designated with the polygon selection tool, respective areas were measured, and area percentages of cortex and medulla were calculated.

### Flow cytometry

Thymuses and spleens were mechanically dissociated in fluorescence-activated cell sorting (FACS) buffer (Ca^2+^/Mg^2+^ free PBS, 1% FBS, and 2mM EDTA) and filtered through a 70μm strainer. Bone marrow (BM) cells were harvested by flushing the femurs and tibias with D-PBS. Cells were spun down and re-suspended in FACS buffer and were stored on ice during processing, staining and analysis. Red blood cells were lysed from spleens and in ACK Lysing Buffer (Gibco). Cells were resuspended in 100 μl of FACS buffer. Fc receptors were blocked with anti-mouse CD16/CD32 (Biolegend) for 15min. Subsequently, cells were stained for surface antigens with directly fluorophore-conjugated antibodies for 30min at 4°C. Hoechst 33342 was used as a viability marker. For intracellular staining, cells were treated using the Foxp3 Transcription Factor Staining Buffer Set (eBiosciences) according to the manufacturer’s instructions, and Zombie UV (Biolegend) was used to measure viability in this case. Cells were assessed on a BD FACSCanto II Cell Analyzer (Salk Institute Flow Cytometry Core Facility) and data were analyzed using FlowJo v10.7.1 software. The antibodies used in flow cytometry are shown in **Supplemental Table 1**. For dendritic cells study, CD11c enrichment fraction was performed from single-cell suspension of thymic tissues using CD11c MicroBeads UltraPure (MACS, Miltenyi Biotec) and LS columns (MACS, Miltenyi Biotec) placed in QuadroMACS Separator (MACS, Miltenyi Biotec), according to manufacturer’s instruction. After positive selection, flow cytometry protocol was followed as usual.

In flow cytometry experiments, live cells were gated first (Hoechst/ZombiUV negative), followed by exclusion of debris using forward and side scatter pulse area parameters (FSC-A and SSC-A). Doublet discrimination was performed using forward and side scatter pulse width parameters (SSC-W and FSC-A, followed by FSC-W and FSC-A). Gating strategies on the population of interest are shown in the figures.

### Isolation and purification of brain immune cells for microglia quantification

Anesthetized WT or *Axl^−/−^Mertk^−/−^* mice (1 mo) were transcardially perfused with ice-cold D-PBS containing Ca^2+^ and Mg^2+^ and brains were promptly dissected out and placed on pre-chilled Petri dishes on ice to extract cortices. All steps were carried out on ice, unless otherwise specified. Cortices were minced with a razor blade, suspended in 6 ml D-PBS in 15-ml tubes for tissue chunks to settle before removal of the supernatant. Single-cell suspensions were prepared following a modified version of the Neural Tissue Dissociation kit from Miltenyi Biotec (130-094-802). Briefly, for each sample, an enzyme mixture 1 containing 1,910 μl buffer Z and 50 μl enzyme P, supplemented with 0.2 μg DNase (Sigma) and an enzyme mixture 2 containing 30 μl Buffer Y and 15 μl of enzyme A were prepared and these enzyme mixtures were kept on ice before usage. The settled tissue pellet was resuspended in mixture 1 and incubated in a water bath at 37°C for 15 min, inverting the tube gently several times every 5 min to resuspend settle cells. Thirty microliters of enzyme mixture 2 was added before mechanical dissociation at room temperature using a fire-polished Pasteur pipette. The cell suspension was further incubated in a water bath at 37°C for 10 min, inverting the tube gently several times every 5 min. Then, fifteen microliters of mixture 2 was added. Samples were subjected to a second round of mechanical dissociation at room temperature with fire-polished Pasteur pipettes with decreasing diameter until no observable tissue pieces remained. The resulting cell suspensions were filtered using 70 μm cell strainers, which were subsequently washed with 10 ml D-PBS supplemented with 0.5% BSA. The cells were centrifuged at 300g for 10 min at 4°C and pellets were suspended in 10 ml 30% isotonic Percoll (GE Healthcare 17-0891-01) diluted in 1× HBSS and then centrifuged again at 700g for 15 min at 4°C with minimum acceleration and braking. The bottom 5 ml of the gradient containing microglia was collected, resuspended, and passed through a 70-μm cell strainer. The cell suspension was washed in ice-cold 1× HBSS for a total volume of 40 ml and pelleted at 300g for 10 min at +4°C. Finally, pellet was resuspended with FACS buffer and flow cytometry protocol was followed as usual.

### RT-qPCR

Total cellular RNA was isolated from whole thymic tissue using TRIzol Reagent (Thermo), according to manufacturer’s instructions. RNA purity and quantity were measured using a nanodrop spectrophotometer (Thermo Scientific). Reverse transcriptase polymerase chain reaction (RT-PCR) was performed using RT Transcriptor First Strand cDNA Synthesis Kit (Roche). Real-time qPCR was performed using SYBR Green PCR Master Mix (Applied Biosystems) on a QuantStudio Q5 system (Applied Biosystem). Analysis was done using comparative Ct method (AACt). *36B4* was used as a control housekeeping gene.

Primer sequences were:

mouse *Il-10*
forward 5’- TGAAGACCCTCAGGATGCG-3’
reverse 5’-TTCACCTGCTCCACTGCCTT-3’
mouse *Il-6*
forward 5’- AGACAAAGCCAGAGTCCTTCAGA-3’
reverse 5’- GCCACTCCTTCTGTGACTCCA-3’
mouse *Il-1b*
forward 5’- CCTCTCCAGCCAAGCTTCC-3’
reverse 5’- CTCATCAGGACAGCCCAGGT-3’
mouse *Nos2*
forward 5’-GCAAGCACCTTGGAAGAGGA-3’
reverse 5’-AGGCCAAACACAGCATACCTG-3’
mouse *Ifng*
forward 5’-CAATCAGGCCATCAGCAACA-3’
reverse 5’-AACAGCTGGTGGACCACTCG-3’
mouse *Tfgb* 5’-AGTGTGGAGCAACATGTGGAA-3’
reverse 5’-CAGCCACTCAGGCGTATCAG-3’
mouse *Il-12p40*
forward 5’-GCTCATGGCTGGTGCAAAG-3’
reverse 5’-TCTGCAGACAGAGACGCCAT-3’
mouse *36b4*
forward 5’-CTCTCGCTTTCTGGAGGGTG-3’
reverse 5’-ACGCGCTTGTACCCATTGAT-3’.

### RNA-seq

Total RNA was isolated from thymuses of 1 month-old mice (3 males and 3 females of WT and *Axl ^-/-^Mertk^-/-^* mice) using the NucleoSpin RNA kit (Macherey-Nagel). The quality of the isolated total RNA was assessed using Agilent TapeStation 4200 and RNA-Seq libraries were prepared with 500 ng total RNA using the TruSeq stranded mRNA Sample Preparation Kit according to the manufacturer’s protocol (Illumina). RNA-seq libraries were multiplexed, normalized and pooled for sequencing. The libraries were sequenced on the HiSeq 2500 system (Illumina) at single read 50bp. Image analysis and base calling were done with Illumina CASAVA-1.8.2. on HiSeq 2500 system and sequenced reads were quality-tested using FASTQC (http://www.bioinformatics.babraham.ac.uk/projects/fastqc). The reads were mapped to the mm10 genome using STAR v2.5.3a (22) and quantified gene expression with HOMER (23) v 4.10.4 using the fragments per kilobase per million mapped reads (FPKM) normalization across exons of the top isoform. Heatmaps show z-score normalized relative expression across conditions for each gene. Differential expression analysis was carried out using HOMER getDiffExpression.pl, using DESeq2 (24) v1.14.1 on top isoform raw exon counts and accounting for sex as a confounding variable. We used a threshold of adjusted p value < 0.05, log2fold > 0.5 to define differentially expressed genes. Bulk RNA-Seq data are publicly available, and have been deposited in the Gene Expression Omnibus (GEO) at the National Center for Biotechnology Information (NCBI) under the submission number GSE192363.

### Creatinine assay in serum samples

Whole blood was collected from the posterior vena cava after mice were euthanized. Blood was allowed to clot by leaving it undisturbed at room temperature during 15-10min. Serum was separated by centrifugation at 2,000rpm for 10min in a refrigerated centrifuge. The serum was immediately transfer to a clean tube and stored at -80°C until tested. For quantitative determination of creatinine in serum, QuantiChrom Creatinine Assay kit (BioAssay Systems) was used following manufacture’s instruction. Optical density was measure on a TECAN Infinite 200 PRO reader.

### Hematology

Whole blood collection was performed by submandibular bleeding. Blood drops were collected into a Microtainer EDTA Tube (BD) and immediately mixed by tapping and inverting the tube to ensure anticoagulation. Samples were kept at room temperature and were tested within 4 hours of collection. Analysis was performed by the UCSD Murine Hematology and Coagulation Core Laboratory, using a Hemavet 950FS Multi-Species Hematology System (Drew Scientific, CT) programmed with mouse setting. All samples were tested by duplicate.

### Differentiation of bone-marrow-derived macrophages

Bone marrow (BM) cells were harvested by flushing the femurs and tibias with D-PBS. Cells were resuspended and filtered using 70 μm cell strainer. BM cells were centrifuged at 300g for 5 min at 4°C. BM cells were resuspended and seeded in differentiation media: Dulbecco’s Modified Eagle Medium (DMEM, Gibco) supplemented with 10% of Fetal Calf Serum (FBS, Corning), 50U/ml of Penicillin-Streptomycin (Gibco) and 30% of L929 supernatant. Fresh differentiation media was added on day 4 and cells were incubated for 3 days more.

### Hemin preparation

Hemin stock solution was prepared as previously described (25): 25mg/mL of hemin (51280, Sigma) in 0.15M NaCl containing 10% NH4OH and stored at -20°C. Hemin was used at a final concentration of 40μM for BMDM culture experiments.

### Data analysis

Statistical analyses were performed using GraphPad Prism software (version 8.0). Statistical analysis and sample size were described in each figure legend. Data are represented as means ± SEM. Values of p < 0.05 were considered to indicate statistical significance.

## Results

### Mer and Axl are expressed by thymic macrophages

**Figure 1:**
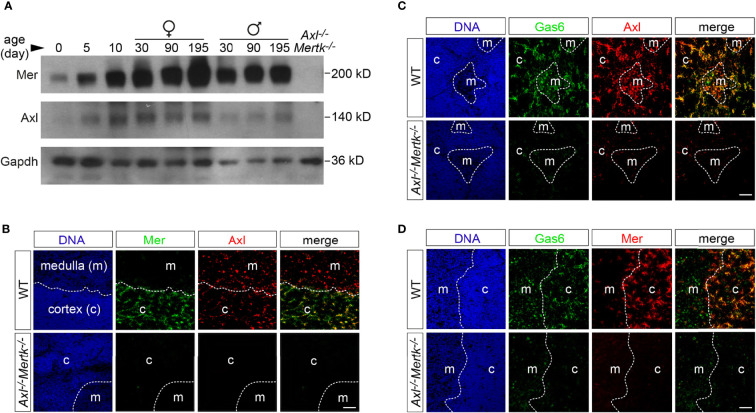
Expression of Axl, Mer, and Gas6 in the thymus. **(A)** Immunoblot analysis of Axl and Mer expression in thymic lysates from WT mice at the designated ages (postnatal days) and sex, with Gapdh as a loading control. *Axl^-/-^Mertk^-/-^* thymic lysate from P30 mice is used as negative control. **(B-D)** Immunohistochemistry of 1 mo WT and *Axl^-/-^Mertk^-/-^* thymic slices with the indicated antibodies. Distinction between cortex (c) and medulla (m) based on nuclear density with Hoechst staining. **(B)** Mer (green) is expressed mainly in the cortex while Axl (red) is expressed in the cortex and medulla in WT mice. All Mer^+^ cells colocalize with Axl. **(C, D)** Gas6 (green) is expressed in the cortex and medulla in Axl^+^ (**C**, red) and Mer^+^ (**D**, red) cells. No Gas6 expression in *Axl^-/-^Mertk^-/-^* mice. **(A–D)** Representative blot and images from n=2-3 mice per genotype. All scale bars, 50μm.

We first examined expression of Tyro3, Axl, and Mer (1) in the mouse thymus during development. As has been noted for TAM expression in many other tissues (1), we did not observe significant Tyro3, Axl, or Mer expression in the embryonic thymus, but detected pronounced up-regulation of Mer and Axl during the first two weeks after birth by western blot (**Figure 1A**). High levels of these receptors were maintained into the adult, with no obvious expression difference noted between males and females across multiple analyses (**Figure 1A**). Very low level Tyro3 expression was detectable in the postnatal thymus upon long exposures of western blots (data not shown). However, we did not detect Tyro3 in any thymic population using either immunohistochemistry (IHC) or flow cytometry, and as detailed below, Tyro3 does not influence the function of thymic macrophages.

At 1 month (mo), when many thymocytes are undergoing apoptosis as a result of ‘death by neglect’ (for non-reactive cells) or negative selection (for auto-reactive cells) (10) and the thymus has reached its maximal cellularity and weight (26), Axl was detected by IHC in both the thymic cortex and medulla, while Mer was more abundant in the cortex (**Figure 1B**). (We used cell density, which is higher in the cortex than the medulla (**Figure 1B**), to distinguish these compartments.) Nearly all Mer^+^ cells in the cortex, where most thymocyte apoptosis is thought to occur (8, 11, 27), also co-expressed Axl (**Figure 1B**). Consistent with previous observations in the spleen, liver, lung, and brain (6, 28), all Axl^+^ cells in the thymus appeared to co-stain for the TAM ligand Gas6 (**Figure 1C**). Gas6 co-localized with cortical Axl^+^Mer^+^ cells and with medullary Axl^+^Mer^-^ cells (**Figures 1C, D**). As has also been observed in other tissues (6, 28), we found that expression of Gas6 protein in the thymus was entirely dependent on TAM receptor expression, as it was lost in *Axl^-/-^Mertk^-/-^* double mutants (2, 20) (**Figures 1C, D**). This loss occurred even though the expression of *Gas6* mRNA is unchanged in the double mutants (6, 28). It is therefore likely that thymic Axl is constitutively bound by Gas6.

**Figure 2:**
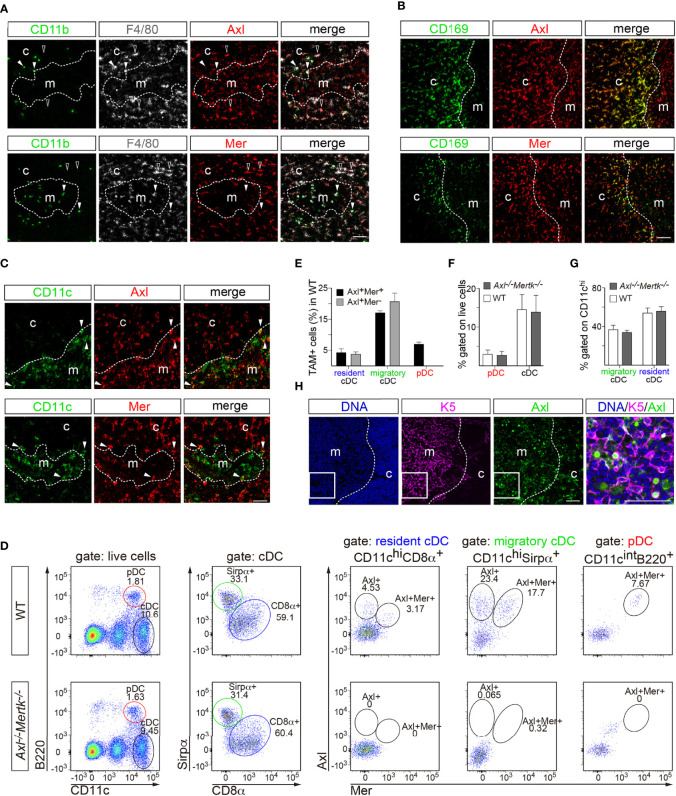
TAM receptor expression in thymic macrophages and dendritic cells. **(A–C, H)** Immunohistochemistry of 1 mo WT thymic slices with the indicated antibodies. Distinction between cortex (c) and medulla (m) based on nuclear density with Hoechst staining. **(A)** F4/80 and CD11b were used to define macrophages. Axl (red) and Mer (red) are expressed by F4/80^+^CD11b^-^ cells (open arrowhead), but not in F4/80^+^CD11b^+^ cells (closed arrowhead). **(B)** CD169^+^ macrophages (green) located in the corticomedullary junction express Axl (red, upper panel) and Mer (red, lower panel). **(C)** CD11c was used as dendritic cell (DC) marker. A low frequency of CD11c^+^ cells (green) express Axl (red, upper panel) and Mer (red, lower panel). **(D–G)** Flow cytometry analysis of an enriched CD11c^+^ thymic single cell suspension at 1 mo. **(D)** Gating strategy to differentiate thymic DC subpopulations and Axl and Mer expression in each: pDC (CD11c^int^B220^+^), cDC (CD11c^hi^ B220^-^), resident cDC (CD11c^hi^ CD8α^+^), migratory cDC (CD11c^hi^ Sirpα^+^). **(E)** Quantification of Axl- and Mer-expressing DCs in WT mice. **(F, G)** Quantification of thymic DC subpopulations in WT and *Axl^-/-^Mertk^-/-^* mice does not reveal significant differences between genotypes. **(H)** K5 was used as medullary thymic epithelial cell (mTEC) marker. A small number of K5^+^ cells (magenta) express Axl (green). Right panel is an enlargement of the indicated area. **(A, B, C, H)** Representative images from n = 2-3 mice per genotype. All scale bars, 50μm. **(E-G)** All graphs are mean +SEM from three independent experiments (n = 3 mice per genotype). Unpaired *t*-test (**F**, **G**).

By far the most prominent cellular loci for thymic Mer and Axl expression were macrophages. Most F4/80^+^ macrophages were positive for both Axl and Mer (**Figure 2A**), with the notable exception of a small number of F4/80^+^ cells in both the cortex and medulla that were also strongly CD11b^+^. At the corticomedullary junction of the thymus, Axl and Mer were also evident in nearly all macrophages that expressed CD169 (sialoadhesin; also seen in metallophilic macrophages in the marginal zone of the spleen) (**Figure 2B**). Only a small number of cells strongly positive for the dendritic cell (DC)-associated antigen CD11c were positive for either Axl or Mer (**Figure 2C**).

Flow cytometry analyses of thymic cells using a set of DC markers allowed us to distinguish cells with the molecular features of resident conventional DCs (CD11c^hi^CD8α^+^ cells), migratory conventional DCs (CD11c^hi^Sirpα^+^ cells), and plasmacytoid DCs (CD11c^int^B220^+^ cells) (**Figure 2D**). Although Mer is generally viewed as a core macrophage marker, Axl^+^Mer^+^ and Axl^+^Mer^-^ cells were equally represented in these DC-like populations, with the exception of plasmacytoid DCs, all of which were Axl^+^Mer^+^ (**Figure 2E**). The frequency of DC subsets in the thymus was not affected by mutation of *Axl* and *Mertk* (**Figures 2D**, **F**, **G**). A fraction of medullary thymic epithelial cells, marked by expression of keratin 5 (K5), were also Axl^+^ (**Figure 2H**).

### Mer and Axl execute apoptotic cell phagocytosis during T cell selection

Among the most important functions of TAM receptors and ligands is the recognition and phagocytic engulfment of ACs (5, 6, 29, 30). This occurs *via* a tripartite bridging arrangement in which the amino terminus of either Gas6 or Pros1 first binds to the plasma membrane phospholipid phosphatidylserine (PtdSer) (1, 31), the most common and most potent of the ‘eat-me’ signals by which dead cells are recognized by phagocytes (4). To complete the bridge, the carboxy terminal domain of Gas6 or Pros1 binds and catalytically activates a TAM receptor expressed on the surface of a phagocyte (3, 29, 31). This mechanism has been most thoroughly studied in phagocytic macrophages (5, 6, 32–34), but also operates in the retinal pigment epithelial cells of the eye (35) and the Sertoli cells of the testes (3, 20). The mutation of different TAM receptor genes in mice, rats, and humans (20, 36, 37) leads to the accumulation of ACs in the spleen (38), lungs (39), brain (30), liver (40), seminiferous tubules (20), retina (41), and other tissues (42).

**Figure 3:**
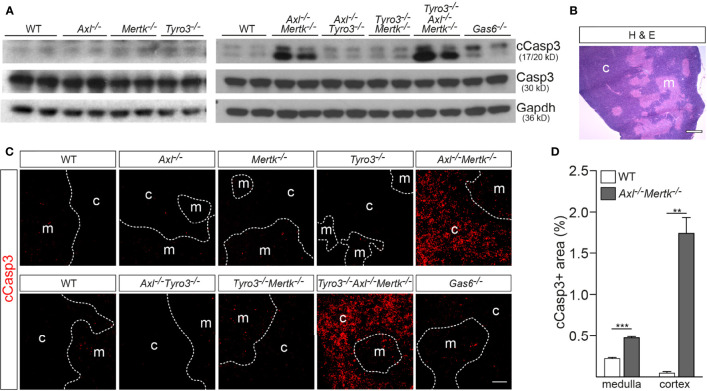
Accumulation of apoptotic cells in the *Axl^-/-^Mertk^-/-^* thymus. **(A)** Immunoblot analysis of cleaved caspase 3 (cCasp3) in thymic lysates from the designated genotypes at 1 mo, with Gapdh as a loading control. cCasp3 levels are markedly elevated in *Axl^-/-^Mertk^-/-^* and *Tyro3^-/-^Axl^-/-^Mertk^-/-^*, and modestly in *Gas6^-/-^*, compared to WT mice. Total levels of Casp3 are comparable between genotypes. **(B)** Representative image of a WT thymic section stained with H&E shows cells densely and loosely packed in the cortex and medulla, respectively. **(C)** Immunohistochemistry of thymic sections from the designated genotypes at 1 mo with cCasp3 shows dramatic accumulation of apoptotic cells in *Axl^-/-^Mertk^-/-^* and *Tyro3^-/-^Axl^-/-^Mertk^-/-^* mainly in the cortex. **(D)** Quantification of cCasp3-positive area in the cortex and medulla from WT and *Axl^-/-^Mertk^-/-^* thymic sections, calculated as the percent of total area. **(A–C)** Representative blot and images from n = 2-3 mice per genotype. Scale bars: **B**, 500μm; **C**, 100μm. **(D)** Graph is mean +SEM from n = 3 WT and n = 2 *Axl^-/-^Mertk^-/-^* mice. Each mouse represents the mean of 3 different sections 100μm apart, and 3 different images per section. **p < 0.01, ***p < 0.001. Unpaired *t*-test.

Even though large numbers of developing T cells undergo apoptosis in the thymus, these ACs are difficult to detect because they have a short half-life and are rapidly phagocytosed (8, 43, 44). We detected low levels of the canonical apoptosis marker cleaved caspase 3 (cCasp3) (45) by western blot in the wild-type (WT) thymus at 1 mo, and these levels were not elevated in *Axl^-/-^*, *Mertk^-/-^*, or *Tyro3^-/-^* single mutants (**Figure 3A**). However, markedly higher cCasp3 was observed in *Axl^-/-^Mertk^-/-^* double mutants and *Tyro3^-/-^Axl^-/-^Mertk^-/-^* triple mutants (**Figure 3A**), at the same time that no elevation in cCasp3 above WT was seen for either *Axl^-/-^Tyro3^-/-^* or *Tyro3^-/-^Mertk^-/-^* double mutants (**Figure 3A**). Thus, in keeping with its low thymic expression, Tyro3 does not play a significant role in phagocytosis in the thymus. Axl is activated exclusively by Gas6, while Mer is equally activated by Gas6 and Pros1 (3). Consistent with this receptor-ligand pairing, cCasp3 levels in the *Gas6^-/-^* thymus were modestly elevated relative to WT, but much less so than in *Axl^-/-^Mertk^-/-^* double mutants (**Figure 3A**). A similar analysis is not possible for *Pros1^-/-^* mice, since these mutants are embryonic lethals (46), but thymic epithelial cells express *Pros1* mRNA (15). All of the above observations together indicate that Axl and Mer, activated by Gas6 and Pros1, act cooperatively and in concert to mediate macrophage phagocytosis of apoptotic thymocytes during T cell selection.

Most thymocyte death by neglect occurs in the thymic cortex (8, 11, 27), and while the site of death by negative selection is to some extent debated, the consensus is that the majority of this apoptotic deletion also occurs in the cortex (47). We examined the distribution of cCasp3^+^ cells across 1 mo thymus sections in the varying genotypes using IHC, and observed a striking disparity between medulla and cortex. As for Figs. 1 and 2, we distinguished these regions based on their relative cell density (cortex higher, medulla lower) (**Figure 3B**). Again, substantial elevation in the number of cCasp3^+^ cells was only seen in the *Axl^-/-^Mertk^-/-^* and *Tyro3^-/-^Axl^-/-^Mertk^-/-^* genotypes, and nearly all of this AC accumulation appeared in the cortex (**Figure 3C**). Quantification revealed that cCasp3^+^ cells occupied a larger fraction of medullary versus cortical area in the WT thymus (**Figure 3D**). However, the incidence of medullary ACs was only elevated ~2-fold relative to WT in *Axl^-/-^Mertk^-/-^* thymus, whereas cortical ACs were ~35-fold more abundant in the double mutant thymus (**Figure 3D**). Note that this striking AC accumulation was seen specifically in the region where macrophages co-express both Axl and Mer (**Figure 1B**). As has been observed in other tissues, it argues that combined signaling through these two RTKs is a potent driver of AC phagocytosis.

The failure of *Axl^-/-^Mertk^-/-^* macrophages to clear apoptotic thymocytes was associated with several additional phenotypes. The total weight of the 1 mo *Axl^-/-^Mertk^-/-^* thymus was increased ~45% relative to WT (**Supplemental Figure 1A**), as was the cellularity (total live cell number) (**Supplemental Figure 1B**). These increases were intrinsic to the thymus, since the body weight of the mice was unchanged (**Supplemental Figure 1C**). Similarly, the relative representation of the cortical and medullary compartments was the same (**Supplemental Figure 1D**). Developing thymocytes progress through a well-described sequence of CD4 and CD8 expression, from CD4^-^CD8^-^ double negative (DN) to CD4^+^CD8^+^ double positive (DP) cells in the cortex, and then to either CD4^+^ or CD8^+^ single positive (SP) T cells in the medulla (48). At 1 mo, the vast majority of thymocytes are CD4^+^CD8^+^ DP cells (48), and these cells were increased in *Axl^-/-^Mertk^-/-^* mutants relative to WT (**Supplemental Figure 1E**). When we analyzed all thymocyte populations for entry into apoptosis using staining with fluorescent Annexin V, which binds to externalized PtdSer (49), we observed that essentially all of this increase in the DP population was due to the failure to clear CD4^+^CD8^+^AnnV^+^ ACs (**Supplemental Figure 1F**).

One possible consequence of the failure to clear early thymic ACs that we have not investigated relates to autoimmunity. The broad spectrum autoimmune disease that develops in *Axl^-/-^Mertk^-/-^* mice (2, 34, 50) is thought to result primarily from the presentation of autoantigens (e.g., ribonucleoproteins) that are derived from uncleared ACs that progress to necrosis, coupled with the highly inflammatory environment that characterizes double mutant immune tissues (5, 7, 51, 52). Our results demonstrate that uncleared ACs dramatically accumulate in the *Axl^-/-^Mertk^-/-^* thymus (**Figure 3C**), and we further observed that this tissue is inflamed relative to WT with respect to elevated mRNA expression of *Il-1b*, *Il-6*, *Il-12p40*, *Ifng*, *Nos2*, and the inflammatory regulators *Il-10* and *Tgfb* (**Supplemental Figure 1G**). However, we also noted that CD5 expression was up-regulated in DP and CD4^+^ SP T cells in the *Axl^-/-^Mertk^-/-^* thymus relative to WT (**Supplemental Figure 1H**), as was the expression of CD69 in CD4^+^ SP T cells (**Supplemental Figure 1I**). Up-regulation of both CD5 and CD69 in CD4^+^ thymocytes normally reflects stronger interactions with self-peptide–MHC complexes (53, 54), and it is therefore possible that some autoreactive CD4^+^ cells, which would normally be phagocytosed by thymic macrophages, escape the thymus as a result of the combined inactivation of Mer and Axl. In addition, we observed that the number of CD4^+^FoxP3^+^ regulatory T cells, deficiencies in which will lead to severe autoimmunity, was also reduced in the *Axl^-/-^Mertk^-/-^* thymus (**Supplemental Figure 1J**).

### Global assessment of TAM receptor regulation of thymic genes

**Figure 4:**
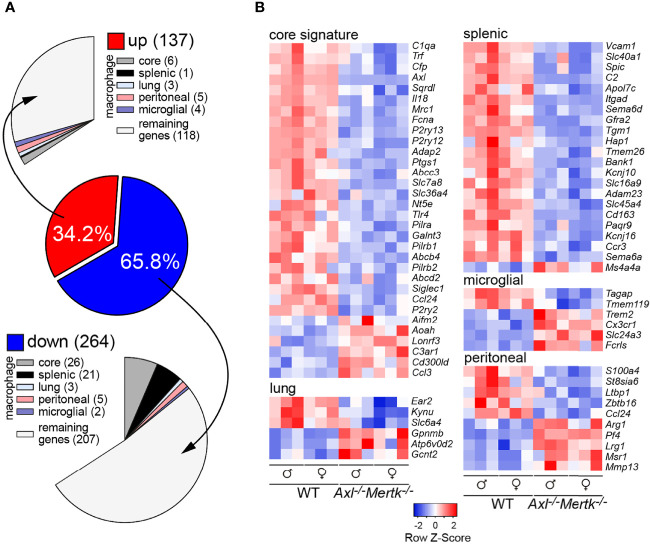
Transcriptomic perturbations in the *Axl^-/-^Mertk^-/-^* thymus. **(A)** Bulk thymic RNA-seq from 1 mo WT and *Axl^-/-^Mertk^-/-^* mice shows 401 differentially expressed genes (DEGs). 137 upregulated and 264 downregulated (adjusted p value < 0.05, log2fold > 0.5) genes in *Axl^-/-^Mertk^-/-^* thymus compared to WT. DEGs between WT and *Axl^-/-^Mertk^-/-^* were compared with the whole set of macrophage core signature genes, and macrophage tissue-specific genes (55). A total of 76 DEGs in the thymus are related to macrophages, with a major contribution from the core signature category (32 DEGs) and the splenic macrophage category (22 DEGs). **(B)** Heatmap of relative expression of DEGs between WT and *Axl^-/-^Mertk^-/-^* thymuses related to macrophages, grouped into the categories of Gautier and colleagues (55) - macrophage core signature genes, lung, splenic, microglial, and peritoneal macrophages genes. n=6 mice per genotype (3 males and 3 females).

To inventory the global consequences of TAM receptor mutation on thymic gene expression, we performed bulk RNA sequencing of all cells in the 1 mo WT and *Axl^-/-^Mertk^-/-^* thymus. We found that approximately two thirds of the thymic mRNAs whose expression was changed as a consequence of TAM receptor mutation were down-regulated (**Figure 4A**), and many of these mRNAs were specifically associated with macrophages (55) (**Figure 4A**). Prominent among down-regulated transcripts were those corresponding to macrophage core signature genes (55), including the complement protein *C1qa*, the purinergic receptors *P2ry2, 12*, and *13*, the Toll-like receptor *Tlr4*, the macrophage cell adhesion molecule *sialoadhesin* (*Siglec1, CD169* in **Figure 2B**), and of course *Axl* (**Figure 4B**). Interestingly, similar down-regulation was also seen for genes normally associated with splenic macrophages (55), including the solute carrier *Ferroportin-1* (*Slc40a1*), the integrin *Itgad*, the hemoglobin scavenger receptor *Cd163*, and the vascular cell adhesion molecule *Vcam1* (**Figure 4B**). Fewer genes annotated as peritoneal macrophage-associated were down-regulated in our RNA-seq survey, and many fewer genes annotated as lung macrophage- or microglial-associated were detected (55) (**Figure 4B**). Importantly, the cohort of down-regulated genes was most prominently linked to macrophage populations that constitutively express high levels of both Mer and Axl and constitutively carry out AC phagocytosis.

Surprisingly, even though our RNA-seq survey was conducted with cells from the thymus, many of the down-regulated genes we identified encode proteins required for the turnover of senescent erythrocytes and the metabolism of hemoglobin and iron – functions specifically carried out by splenic RPMs. These include the transcription factor *Spic* (Spi-C), which is highly enriched in and absolutely required for the differentiation of RPMs (15, 16, 55) (**Figure 4B**). Also down-regulated in the *Axl^-/-^Mertk^-/-^* thymus were the immunoregulator *Sema6d* (Semaphorin 6D), the Neurturin receptor *Gfra2*, the transglutaminase *Tgm1*, the solute transporter *Slc16a9*, and the inwardly rectifying K^+^ channel *Kcnj10*, all of which are also normally enriched in or specific to RPMs (15, 55). Down-regulated genes tied to iron metabolism include the aforementioned *Slc40a1* and *Cd163*. This unexpected and unusually broad dysregulation of genes associated with RPMs and the handling of heme and hemoglobin is especially relevant to the clinical phenotypes described below.

### TAM mutation leads to a body-wide deficit in tissue resident macrophages

**Figure 5:**
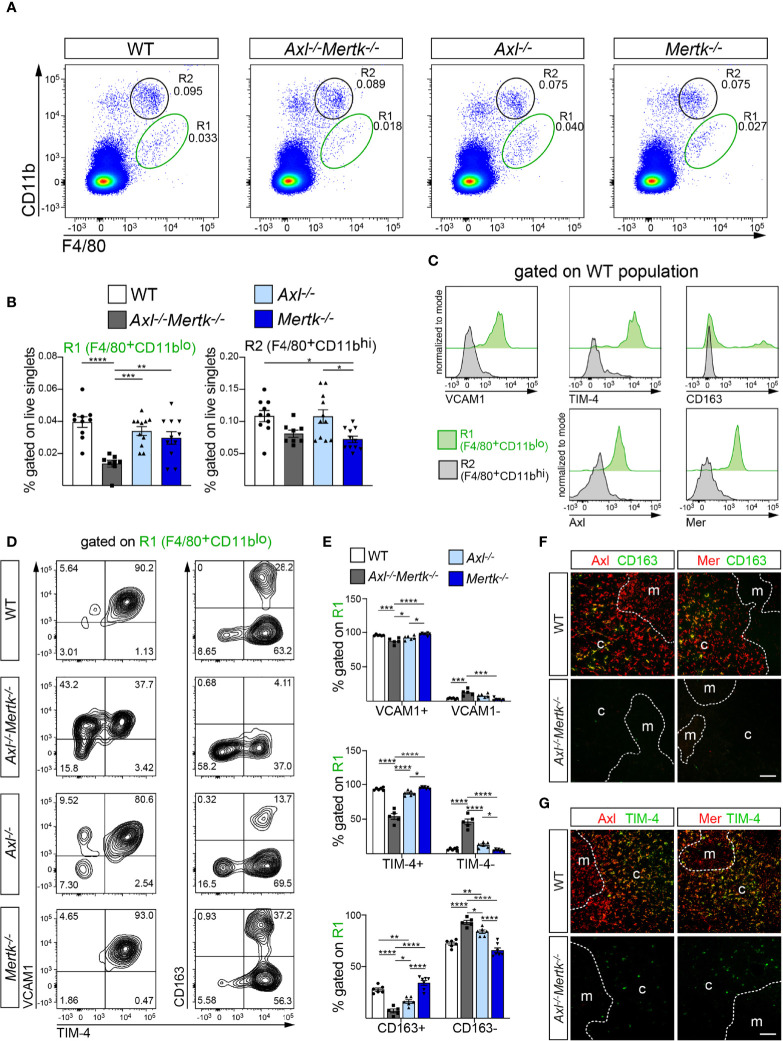
Specific depletion of highly phagocytic macrophages from the *Axl^-/-^Mertk^-/-^* thymus. **(A–E)** Single cell suspensions from 1 mo WT, *Axl^-/-^Mertk^-/-^*, *Axl^-/-^*, and *Mertk^-/-^* thymuses were stained and analyzed by flow cytometry. **(A, B)** Representative cytometry plots **(A)** and quantification **(B)** of F4/80^+^CD11b^lo^ (R1) and F4/80^+^CD11b^hi^ (R2) cells. Frequencies calculated as the percent of live singlet cells. **(C)** Representative histograms indicating macrophage marker expression (VCAM1, TIM-4, CD163, Axl and Mer) in R1 (green) and R2 (grey) populations from WT thymus. VCAM1, TIM4, CD163, Axl and Mer are exclusively expressed in R1. **(D)** Representative cytometry plots of VCAM1, TIM-4 and CD163 expression on F4/80^+^CD11b^lo^ (R1) cells. **(E)** Frequencies of VCAM1^+^ and VCAM1^-^ (top panel), TIM-4^+^ and TIM-4^-^ (middle panel) and CD163^+^ and CD163^-^ (lower panel), calculated as the percent of R1. **(F, G)** Immunohistochemistry of 1 mo WT and *Axl^-/-^Mertk^-/-^* thymic sections illustrate Axl (red) and Mer (red) expression in CD163^+^ (**F**, green) and TIM-4^+^ (**G**, green) cells in WT mice. Note marked reduction of CD163 and TIM-4 expression in *Axl^-/-^Mertk^-/-^* thymus. Distinction between cortex **(c)** and medulla (m) based on nuclear density with Hoechst staining. **(F, G)** Representative images from n = 2-3 mice per genotype. All scale bars, 100μm. **(B, E)** Graphs are mean ± SEM from at least 3 independent experiments. Each data point represents a separate mouse: **(B)** n = 8-11 mice per genotype, **(E)** n = 5-7 mice per genotype. *p < 0.05, **p < 0.01, ***p < 0.001 and ****p <0.0001. One-way ANOVA followed by Tukey’s multiple comparison test.

The widespread loss of macrophage core signature genes in the *Axl^-/-^Mertk^-/-^* thymus led us to examine the effects of TAM mutation on the representation of distinct macrophage populations in the thymus and also in other tissues. We used flow cytometry to analyze thymic cells from WT, *Axl^-/-^*, *Mertk^-/-^*, and *Axl^-/-^Mertk^-/-^* mice at 1 mo using antibodies against the tissue macrophage marker F4/80 (56) and the integrin CD11b, and identified two populations – F4/80^+^CD11b^lo^ (designated R1) and F4/80^+^CD11b^hi^ (designated R2), which have been described previously (14) (**Figure 5A**). Unexpectedly, the population size of the R1 macrophages, which have been shown to be exceptionally phagocytic in the thymus and liver (14, 57), was dramatically reduced specifically in *Axl^-/-^Mertk^-/-^* mice (**Figures 5A**, **B**). The large R1 reduction we detected was stable over time, and was fully maintained in the adult *Axl^-/-^Mertk^-/-^* thymus at 3 mo (**Supplemental Figure 2A**). Statistically significant diminution of the 1 mo R1 population was not seen in either *Axl^-/-^* or *Mertk^-/-^* single mutants (**Figures 5A**, **B**). The R2 population was less affected at 1 mo, although modest reductions were seen upon *Mertk* mutation. Importantly, we observed that WT R1 macrophages specifically expressed both Axl and Mer, while R2 cells did not (**Figure 5C**). Consistent with their vigorous activity as AC phagocytes, only R1 macrophages also expressed the phagocytic receptor TIM-4 (**Figure 5C**). Similarly, markers tied to highly phagocytic RPMs, including the important hemoglobin-haptoglobin scavenger receptor complex CD163 and the adhesion sialoglycoprotein VCAM1, were also expressed in R1 but not in R2 cells (**Figure 5C**). Given the phenotypic similarities between R1 macrophages in the thymus and F4/80^+^CD11b^lo^VCAM1^+^ RPMs in the spleen (16, 58), the depletion of the R1 population in *Axl^-/-^Mertk^-/-^* mice almost certainly contributes to the down-regulation of *Cd163*, *Slc40a1*, *Spic*, and other RPM-associated genes that we quantified in our RNA-seq survey (**Figure 4B**).

As noted above, nearly all R1 macrophages are VCAM1^+^TIM-4^+^ in the WT thymus. While VCAM1 expression was only modestly reduced in *Axl^-/-^Mertk^-/-^* mice, approximately half of the double mutant R1 population lost expression of TIM-4 (**Figures 5D**, **E**). Similarly, while CD163^+^ cells accounted for ~30% of the WT thymic R1 population, the expression of this marker was nearly undetectable in the same population in the *Axl^-/-^Mertk^-/-^* thymus (**Figures 5D**, **E**). This deficit in cells expressing key phagocytic receptors is again consistent with the large number of ACs that accumulate in the *Axl^-/-^Mertk^-/-^* thymus. IHC analyses indicated that while CD163 was expressed by a subset of Axl^+^Mer^+^ cells in the cortex (**Figure 5F**), TIM-4 was expressed by essentially all of these cells (**Figure 5G**).

**Figure 6:**
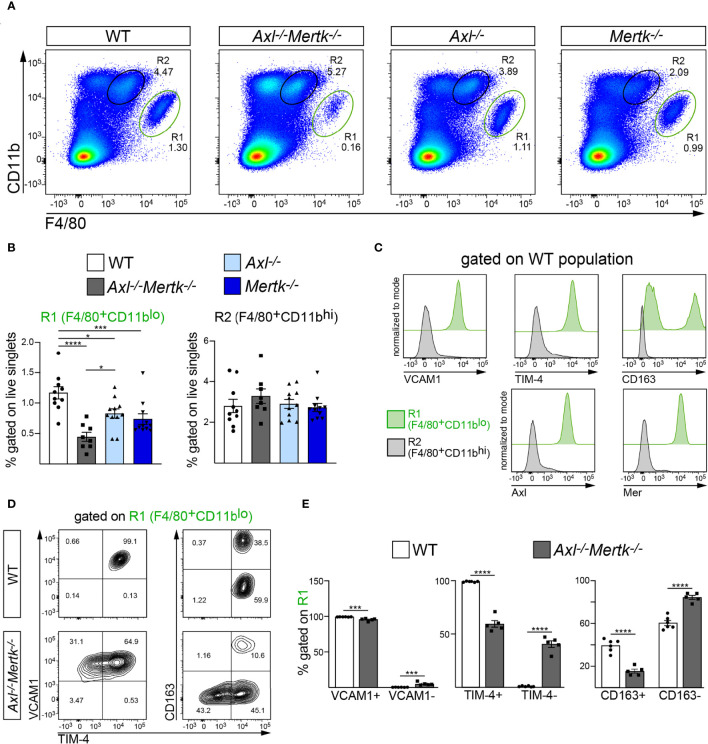
Specific depletion of red pulp macrophages from the *Axl^-/-^Mertk^-/-^* spleen. **(A–E)** Single cell suspension from 1 mo WT, *Axl^-/-^Mertk^-/-^*, *Axl^-/-^*, and *Mertk^-/-^* spleens were stained and analyzed by flow cytometry. **(A, B)** Representative cytometry plots **(A)** and quantification **(B)** of F4/80^+^CD11b^lo^ (R1, red pulp macrophages) and F4/80^+^CD11b^hi^ (R2) cells. Frequencies calculated as the percent of live singlet cells. **(C)** Representative histograms indicating macrophage marker expression (VCAM1, TIM-4, CD163, Axl and Mer) in R1 (green) and R2 (grey) populations from WT spleen. VCAM1, TIM4, CD163, Axl and Mer are exclusively expressed in R1. **(D)** Representative cytometry plots of VCAM1, TIM-4 and CD163 expression on F4/80^+^CD11b^lo^ (R1) cells, in WT and *Axl^-/-^Mertk^-/^* ^-^ mice. **(E)** Frequencies of VCAM1^+^ and VCAM1^-^ (left panel), TIM-4^+^ and TIM-4^-^ (middle panel) and CD163^+^ and CD163^-^ (right panel), calculated as the percent of R1. **(B, E)** Graphs show mean ± SEM from at least 3 independent experiments. Each data point represents a separate mouse: **B**, n=8-11 mice per genotype; **E**, 5-6 mice per genotype. *p < 0.05, ***p < 0.001 and ****p <0.0001. **B**, One-way ANOVA followed by Tukey’s multiple comparison test. **E**, Unpaired *t*-test.

Very importantly, these deficits in the thymic R1 population were also seen in other tissues. In the 1 mo spleen, where the major R1 population is comprised of RPMs (16, 58), we quantified a >60% reduction in this population in *Axl^-/-^Mertk^-/-^* mice relative to WT (**Figures 6A**, **B**). Splenic R1 macrophages were also reduced in *Axl^-/-^* and *Mertk^-/-^* single mutants, albeit more modestly so (**Figure 6B**). As seen in the thymus, the *Axl^-/-^Mertk^-/-^* R1 reduction in the spleen was maintained at 3 mo (**Supplemental Figure 2B**). As also seen in the thymus, all splenic R1 macrophages were strongly positive for Axl and Mer, and also for VCAM1 and TIM-4, whereas splenic R2 cells were not (**Figure 6C**). A large fraction of splenic R1 cells were similarly positive for the hemoglobin receptor CD163, whereas R2 cells were negative (**Figure 6C**). Within the depleted R1 population of the *Axl^-/-^Mertk^-/-^* spleen, the expression of both TIM-4 and CD163 was, as in the thymus, very substantially reduced at both 1 mo (**Figures 6D**, **E**) and 3 mo (**Supplemental Figure 2C**).

Bone marrow macrophages (BMM) share the same developmental origin and display many of the phenotypic features of splenic RPMs. These macrophages are also defined as F4/80^+^CD11b^lo^VCAM1^+^ cells, express Mer and CD163, and require the transcription factor Spi-C for their development (58, 59). Using the same gating strategy that we employed in the thymus and spleen, we again quantified a marked reduction in the BMM R1 macrophage population in *Axl^-/-^Mertk^-/-^* mice relative to WT (**Supplemental Figures 3A**, **B**). We found that this R1 population normally expressed not only Mer but also Axl, and that in close correspondence with the thymus and spleen, it was also strongly positive for TIM-4 and VCAM1 and similarly fractionally positive for CD163 (**Supplemental Figure 3C**).

These R1 macrophage populations in the thymus, splenic red pulp, and bone marrow are distinctive. First, they highly express both Mer and Axl. (The Kupffer cells of the liver, which are also F4/80^+^CD11b^lo^ and highly phagocytic, are a similar population (40).) This is in contrast to most TRMs, which are strongly positive for Mer but only very weakly positive for Axl in the absence of stimulation (15, 39). Second, and again in contrast to most TRMs, the Axl^+^Mer^+^ cells of the thymus, spleen, and bone marrow all carry out routine (continuous) phagocytosis that extends from months to years. The R1 deficits described above may be specific to these important Axl^+^Mer^+^ highly phagocytic macrophages, since we did not detect a significant difference in the representation of brain microglia, which are Axl^-^Mer^+^ (15, 28, 30) and are not engaged in continuous routine phagocytosis (30), between WT and *Axl^-/-^Mertk^-/-^* mice (**Supplemental Figures 3D**, **E**). As noted above, we also did not detect a substantial reduction in the expression of microglia-annotated genes by RNA-seq (**Figure 4B**). Together, these findings indicate that mutation of Axl and Mer leads not only to the loss of these two important phagocytic receptors, but also to a large decrease in the number of highly phagocytic F4/80^hi^CD11b^lo^ macrophages within multiple tissues. Even further, it results in markedly diminished expression of other phagocytic receptor systems – including those anchored by TIM-4 and CD163 – in the reduced number of R1 macrophages that remain in all of these double mutant tissues.

### TAM mutation results in dysregulation of iron metabolism and anemia

**Figure 7:**
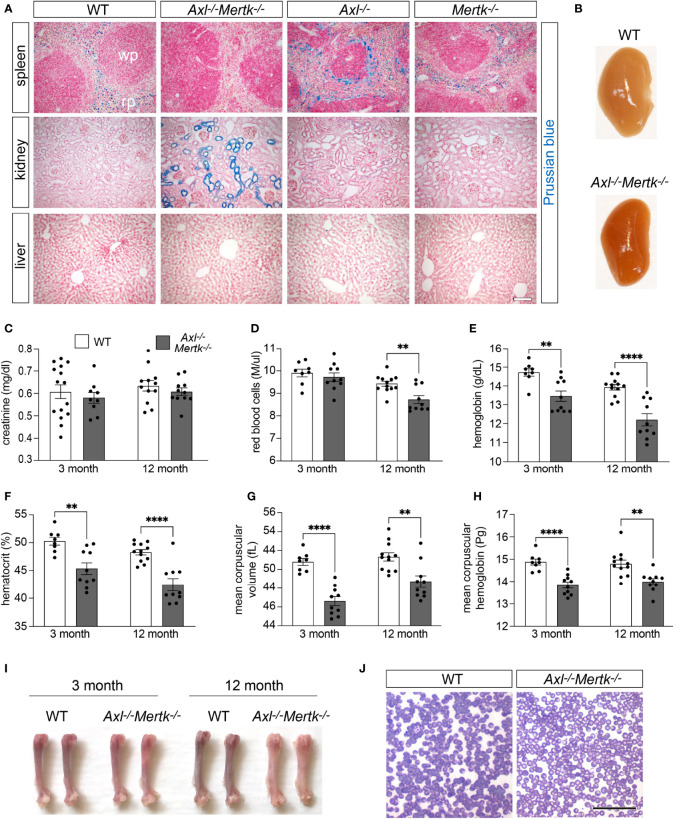
Perturbations in iron metabolism in *Axl^-/-^Mertk^-/-^* mutants. **(A)** Representative images of Prussian blue staining (iron detection) in spleen, kidney, and liver sections from 3 mo WT, *Axl^-/-^Mertk^-/-^*, *Axl^-/-^*, and *Mertk^-/-^* mice. The lack of Prussian blue staining in the splenic red pulp (RP), and the staining of the kidney tubules in *Axl^-/-^Mertk^-/-^* mice indicate a defect in iron handling in the double mutants. **(B)** Representative images of kidneys from 3 mo WT and *Axl^-/-^Mertk^-/-^* perfused mice, show differences in kidney coloration. **(C)** Serum creatinine, **(D)** red blood cell concentration, **(E)** hemoglobin concentration, **(F)** hematocrit, **(G)** mean corpuscular volume, and **(H)** mean corpuscular hemoglobin, measured in adult (3 mo) and mature (12 mo) WT and *Axl^-/-^Mertk^-/-^* mice. **(I)** Representative image of femur bones from two separate mice per group. *Axl^-/-^Mertk^-/-^* mice show pale bone marrow compared to WT at 12 mo. **(J)** Representative image of blood smears at 12 mo (rapid panoptic staining) shows pale red blood cells in *Axl^-/-^Mertk^-/-^* mice. **(A, B, J)** Representative images from n=2-3 mice per genotype. Scale bars: **A**, 100 μm and **J**, 50μm. **(C–H)** Graphs are mean ± SEM from n=8-15 mice per genotype. Each data point represents one separate mouse **p < 0.01 and ****p <0.0001. Unpaired *t*-test.

The unexpected observations detailed above, including the reduction in CD163 and TIM-4 expression in the depleted R1 populations of the *Axl^-/-^Mertk^-/-^* thymus and spleen, suggested that TAM receptor mutants may exhibit unanticipated phenotypes related to their observed deficits in routine phagocytosis. We therefore examined the possible consequences of RPM depletion in the *Axl^-/-^Mertk^-/-^* spleen. Among the best-studied functions of RPMs is the phagocytosis of senescent and damaged erythrocytes, and the metabolism of their hemoglobin- and iron-laden contents (17–19). Indeed, these splenic macrophages are among the most active mammalian phagocytes, since in humans exhausted erythrocytes are generated at a rate of ~2 x 10^6^/second (60). RPM deficiencies and the dysregulation of erythrocyte phagocytosis have multiple sequelae for iron handling (16, 61). We therefore analyzed WT, *Axl^-/-^*, *Mertk^-/-^*, and *Axl^-/-^Mertk^-/-^* spleens using the potassium ferrocyanide Perls Prussian blue reaction, a sensitive histological stain for protein-bound ferric iron (62). We detected abundant (normal) Prussian blue deposits in the red pulp of WT, *Axl^-/-^*, and *Mertk^-/-^* spleens, corresponding to macrophage sequestration of Fe^3+^ iron (**Figure 7A**). In marked contrast, these iron deposits were undetectable in the *Axl^-/-^Mertk^-/-^* red pulp (**Figure 7A**). Accumulation of ferric iron in the liver, another important tissue with respect to iron homeostasis (63), was not observed in any genotype (**Figure 7A**).

The absence of significant iron sequestration in the *Axl^-/-^Mertk^-/-^* spleen is almost certainly due to two deficiencies, the first of which is the defective clearance of senescent erythrocytes. As has been seen in other mouse mutants that lack a normal complement of RPMs (61), we hypothesized that these uncleared erythrocytes eventually rupture and release their hemoglobin and heme into the *Axl^-/-^Mertk^-/-^* circulation. In this setting, and also following intravascular hemolysis and induced hemolytic anemia, iron has been shown to be retained by the epithelial cells of the proximal tubules of the kidney (64, 65). Consistent with these earlier observations, we found that anomalous Prussian blue staining appeared specifically in the proximal tubules of the *Axl^-/-^Mertk^-/-^* kidney (**Figure 7A**). The abnormal deposition of iron in the kidneys of the *Axl^-/-^Mertk^-/-^* double mutants resulted in an obvious increased redness of the entire organ (**Figure 7B**). Although high levels of iron can compromise renal function (64), *Axl^-/-^Mertk^-/-^* mice did not display a significant deficit in kidney filtration function as measured by creatinine levels in the circulation (**Figure 7C**).

The second deficiency that contributes to iron deposition in the *Axl^-/-^Mertk^-/-^* kidney is the widespread loss of macrophage CD163 documented above (**Figures 5D**, **E**). This receptor is one of the principal scavenging systems for cell-free hemoglobin (Hb). Free hemoglobin is bound by haptoglobin (Hp), and Hb-Hp complexes are transported to macrophages, where they are recognized by macrophage CD163 and then internalized by endocytosis (66). Both acute hemolysis and the absence of Hp (in *Hp^-/-^* mice) also result in the deposition of ferric iron in the proximal tubules of the kidneys (65, 67), as we have observed in *Axl^-/-^Mertk^-/-^* mice (**Figure 7A**).

The activities of the macrophages that are reduced in the splenic red pulp and the bone marrow of *Axl^-/-^Mertk^-/-^* mice, including their ability to recycle iron from senescent red blood cells, are also essential to erythropoiesis (68). We therefore asked whether the double mutants exhibited deficits in erythropoiesis by measuring multiple hematological parameters in the circulation. We found that hemoglobin concentration (**Figure 7E**), hematocrit (**Figure 7F**), mean corpuscular volume (**Figure 7G**), and mean corpuscular hemoglobin (**Figure 7H**) were all consistently reduced in *Axl^-/-^Mertk^-/-^* mice at both 3 mo and 1 year. At the latter time, erythrocyte concentration was also reduced in the double mutants (**Figure 7D**), consistent with a possible worsening of erythropoiesis with age in these mice. In keeping with this possibility, the large bones of *Axl^-/-^Mertk^-/-^* mice were significantly paler than their WT counterparts at 1 year (**Figure 7I**), and individual *Axl^-/-^Mertk^-/-^* erythrocytes were far less intensely stained in blood smears owing to the deficit in mean corpuscular hemoglobin (**Figure 7J**). Together, these deficits, which have not been previously described, indicate that *Axl^-/-^Mertk^-/-^* mice suffer from hypochromic microcytic anemia, which most commonly results from decreased iron reserves in the body.

### Continuous death of phagocytic macrophages in TAM mutant tissues

**Figure 8:**
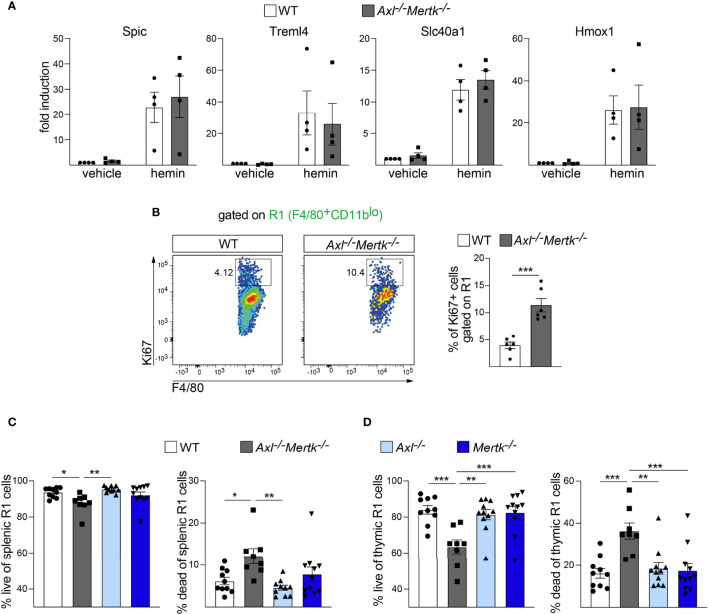
Normal heme regulation but continuous birth and death in *Axl^-/-^Mertk^-/-^* macrophages. **(A)** Relative gene expression in WT versus *Axl^-/-^Mertk^-/-^* bone-marrow-derived macrophages stimulated *in vitro* for 4 days with hemin (40μM) or vehicle. Graphs are means ± SEM from 4 independent experiments. **(B)** Representative cytometry plots (left) and quantification (right) of Ki67^+^ cells gated on the splenic F4/80^+^CD11b^lo^ (R1) macrophage population from 1 mo WT and *Axl^-/-^Mertk^-/-^* mice. Frequencies calculated as the percent of R1. Graph is mean ± SEM from 2 independent experiments, n=6 mice per genotype. Each data point represents a separate mouse. **(C)** The splenic F4/80^+^CD11b^lo^ (R1) macrophage populations from 1 mo WT, *Axl^-/-^*, *Mertk^-/-^*, and *Axl^-/-^Mertk^-/-^* mice were further segregated into Hoechst^-^ (live) and Hoechst^+^ (dead) populations and quantified by flow cytometry. **(D)** The thymic F4/80^+^CD11b^lo^ (R1) macrophage populations from 1 mo WT, *Axl^-/-^*, *Mertk^-/-^*, and *Axl^-/-^Mertk^-/-^* mice were further segregated into Hoechst^-^ (live) and Hoechst^+^ (dead) populations and quantified by flow cytometry. Graphs in **C** and **D** show mean ± SEM from at least 3 independent experiments. Each data point represents a separate mouse (n=8-11 mice per genotype). **(A)** Two-Way ANOVA, **(B–D)** One-way ANOVA followed by Tukey’s multiple comparison test. *p < 0.05, **p < 0.01, and ***p < 0.001.

The pleiotropic deficits in RPM number and function we detected in the *Axl^-/-^Mertk^-/-^* spleen were not due to an inability of the progenitors of these cells to respond to Fe^3+^-bound heme (hemin), which is a key signal that drives their differentiation. It is well-established that the development of RPMs, from either monocytes *in vivo* or bone-marrow derived macrophages (BMDM) *in vitro*, is dependent on heme-mediated induction of the transcription factor Spi-C and other differentiation drivers (25, 58, 69). When we assessed the ability of hemin to induce expression of mRNAs encoding the RPM markers *Spic*, *Treml4*, *Slc40a1*, and *Hmox1* in BMDM, we detected no difference in the level of induction of these mRNAs in WT versus *Axl^-/-^Mertk^-/-^* cells (**Figure 8A**).

RPM deficits in *Axl^-/-^Mertk^-/-^* mice instead appear to result from one of the best known functions of Axl and Mer – the ability of these RTKs to function as cell survival factors (1, 70, 71). When we assayed splenic R1 (F4/80^+^CD11b^lo^) macrophages for the fraction of cells expressing the cell proliferation marker Ki67 (72), we were surprised to detect ~3-fold more Ki67^+^ cells in *Axl^-/-^Mertk^-/-^* as compared to WT mice (**Figure 8B**). Remarkably, we found that this elevated Ki67 expression was coupled to the continuing death of RPMs in the *Axl^-/-^Mertk^-/-^* spleen. At 1 mo, the number of dead F4/80^+^CD11b^lo^ (R1) macrophages was elevated 2-fold in *Axl^-/-^Mertk^-/-^* mice relative to WT (**Figure 8C**). Consistent with TAM expression in this population and with the results presented above, this increase was only seen in the *Axl^-/-^Mertk^-/-^* spleen, and did not develop in *Axl^-/-^* or *Mertk^-/-^* single mutant mice (**Figure 8C**). Although some of these dead cells may accumulate due to defective phagocytosis, the continuing elevated proliferation of the splenic *Axl^-/-^Mertk^-/-^* R1 population argues that Axl and Mer also function as survival factors for these cells. We similarly measured a 2-fold steady-state increase in the level of dead F4/80^+^CD11b^lo^ macrophages in the double mutant thymus, but not in the *Axl^-/-^* or *Mertk^-/-^* thymus, at 1 mo (**Figure 8D**). Together these results indicate that the population of highly phagocytic macrophages is continuously dividing and dying in *Axl^-/-^Mertk^-/-^* tissues, without ever displaying a normal complement of end-stage phagocytic effectors.

## Discussion

The TAM receptors Mer and Axl, in concert with their ligands Gas6 and Pros1, are firmly established as critical mediators of the phagocytosis of ACs by macrophages (1, 4–6) and of the macrophage clearance of cellular debris and components that display the apoptotic eat-me signal PtdSer (28, 35, 73). Indeed, this tripartite apparatus is among the most powerful AC clearance pathways in the immune system (4). Remarkably, however, the role that TAM signaling might play during normal postnatal T cell selection in the developing thymus, when large numbers of ACs are generated, has not been previously investigated. Although earlier analyses have found that TAM receptor expression is required to handle the very large number of cells that are artificially killed in the thymus after high-dose administration of dexamethasone (5, 33), our results demonstrate that Mer and Axl are together required for the clearance of the apoptotic thymocytes that are generated naturally during the normal course of postnatal T cell differentiation and maturation.

While the dramatic accumulation of ACs that we document in the *Axl^-/-^Mertk^-/-^* thymus is consistent with earlier work in other tissues, our new results indicate that this pile-up of dead cells, in both the thymus and elsewhere, is not simply due to the loss of these phagocytic receptors alone. We additionally find that *Axl* and *Mertk* double mutation leads to the depletion of >50% of highly phagocytic F4/80^+^CD11b^lo^ macrophages (14), which execute PtdSer-dependent AC engulfment in the thymus, spleen, bone marrow, and probably other tissues as well. Our results suggest that this depletion, which has not been previously detected, is tied to the well-described ability of Axl and Mer to functions as survival drivers for many different cancer cells (71, 74, 75). Exacerbating this deficit even further, we find that the expression of TIM-4, CD163, and other engulfment and scavenger mediators is dramatically reduced in the depleted population of F4/80^+^CD11b^lo^ cells that remains. Consistent with this observation is the fact that (a) CD163 expression has previously been found to be specifically associated with Mer expression in cultured ‘M2c-like’ human macrophages (76), and (b) enhanced Mer expression in mouse macrophages has previously been seen to be associated with enhanced expression of the phagocytosis mediator C1q (77, 78), whose mRNA was reduced in the *Axl^-/-^Mertk^-/-^* thymus in our RNA-seq survey. Although our findings of TAM regulation of multiple phagocytosis mediators appear to extend to many other highly phagocytic Axl^+^Mer^+^ macrophages, in earlier studies we and others found that TIM-4 expression in peritoneal macrophages, which express Mer but little or no Axl (15, 39), is unaffected by *Mertk^-/-^* single mutation (79) or *Axl^-/-^Mertk^-/-^* double mutation (29). In any event, it is clear that with respect to phagocytosis, many of the macrophages in *Axl^-/-^Mertk^-/-^* mutants suffer from a previously unrecognized ‘triple whammy’ of engulfment deficits.

The tissue-resident macrophages that are specifically depleted upon combined mutation of *Mertk* and *Axl* are unusual. They are distinguished by their high steady-state expression of both receptors, and by the continuous use of the TAM system to phagocytically clear ACs and PtdSer-displaying organelles over months and years. For example, a new bolus of tens of billions of exhausted erythrocytes (whose lifespan is ~120 days) is cleared by splenic RPMs during every day of mammalian life (80, 81). Similarly, at every cycle of erythropoiesis to replace these red blood cells, the nuclei of newly born erythrocytes, which are surrounded by a PtdSer-rich membrane, are extruded and phagocytically engulfed by resident macrophages in the mammalian bone marrow (82). And as demonstrated above, the apoptotic thymocytes generated during T cell selection in the thymus are continuously cleared by tissue-resident macrophages. In contrast to most peritoneal macrophages, microglia, and blood monocytes, which express abundant Mer but only very low levels of Axl (15, 28, 55), the R1 macrophages that perform continuous PtdSer-dependent phagocytosis in the thymus, spleen, and bone marrow are strongly positive for both TAM receptors. Alveolar macrophages of the lungs, which must carry out this phagocytosis with nearly every inspiration, are also strongly positive for both Axl and Mer (39); as are Kupffer cells of the liver (40), which are also continuously active phagocytes (83).

The multifaceted deficits in phagocytic cells and phagocytic receptor systems that develop in the *Axl^-/-^Mertk^-/-^* mutants – including the pronounced pile-up of ACs and the elevated expression of multiple inflammatory cytokines - have a series of previously unrecognized consequences for these mice. These include the marked perturbations in iron metabolism documented above. Our data indicate that *Axl^-/-^Mertk^-/-^* mice do not efficiently clear senescent erythrocytes or normally sequester heme-complexed iron (probably *via* CD163) in the spleen. They instead display ferric iron sequestration in the kidney, exhibit reduced hemoglobin, hematocrit, mean corpuscular volume, and mean corpuscular hemoglobin in the circulation, and are very broadly anemic. Although mouse mutants in the *Axl* and *Mertk* genes were first generated over 20 years ago (2, 20, 84), none of the above phenotypes have heretofore been reported. These mutants therefore continue to offer surprises. The fact that the Axl^+^Mer^+^F4/80^+^CD11b^lo^ highly phagocytic macrophage population is continuously churning (dividing and dying) and severely reduced in many, perhaps all, tissues in the double mutants has also not been recognized heretofore. It suggests that Axl and Mer are coordinately required for the maintenance of these important cells throughout life.

## Data availability statement

The datasets presented in this study can be found in online repositories. The names of the repository/repositories and accession number(s) can be found below: NCBI Gene Expression Omnibus (GEO) database under the accession number GSE192363.

## Ethics statement

The animal study was reviewed and approved by Salk Institute Institutional Animal Use and Care Committee.

## Author contributions

LJ-G designed and performed experiments in all sections of the paper, CM performed initial analyses of TAM receptor expression in the postnatal thymus, PB assisted with histology and immunohistochemistry, YH enabled both microglia isolation and immunohistochemical studies, MNS performed bioinformatic analyses, GL conceived the study and wrote the initial draft of the paper. All authors contributed to the article and approved the submitted version.

## Funding

Supported by grants from the US National Institutes of Health (RF1 AG060748 and R01 AI101400 to GL, and P30 CA014195 and S10-OD023689 to the Salk Institute), the Leona M. and Harry B. Helmsley Charitable Trust (to the Salk Institute); and by postdoctoral fellowships from the Fundación Alfonso Martín Escudero and the Nomis foundation (to LJ-G). The authors declare that this study received funding from Ferring Pharmaceuticals. The funder was not involved in the study design, collection, analysis, interpretation of data, the writing of this article or the decision to submit it for publication.

## Acknowledgments

We thank Joseph Hash for technical assistance, Caz O’Connor for advice on flow cytometry analysis and experimental design and for management of the Salk Institute flow cytometry core, Nasun Hah for assistance with RNA sequencing, Antonio Castrillo for advice on macrophage characterization, and members of the Lemke lab and the Nomis Center for discussions.

## Conflict of interest

The authors declare that the research was conducted in the absence of any commercial or financial relationships that could be construed as a potential conflict of interest.

## Publisher’s note

All claims expressed in this article are solely those of the authors and do not necessarily represent those of their affiliated organizations, or those of the publisher, the editors and the reviewers. Any product that may be evaluated in this article, or claim that may be made by its manufacturer, is not guaranteed or endorsed by the publisher.

## Supplementary material

Supplementary Figure 1 Cellular, inflammatory, and autoimmune perturbations in the *Axl^-/-^Mertk^-/-^* thymus. Increased **(A)** thymus weight and **(B)** cellularity in *Axl^-/-^Mertk^-/-^* compared to WT mice at 1 mo. No change in **(C)** body weight is evident between genotypes. **(D)** Representative images of 1 mo thymic sections stained with H&E (left), and quantification (right) of the ratio cortex (c) and medulla (m) represented as the percent of total area. **(E)** Quantification of TCRβ^+^ thymocyte subpopulations gated on live singlet cells from 1 mo WT and *Axl^-/-^Mertk^-/^* ^-^ mice, analyzed by flow cytometry. **(F)** Thymocytes assayed for apoptosis by flow cytometry. Propidium iodide (PI) positive cells were discarded (dead cells). Only viable (PI^-^) singlet TCRβ^+^ thymocytes were segregated and analyzed based in their AnnexinV (AnnV) staining: TCRβ^+^ AnnV^+^ thymocytes (viable, early apoptotic) and TCRβ^+^ AnnV^-^ thymocytes (viable, non-apoptotic). **(E, F)** Data are represented as number of TCRβ^+^ thymocytes in 10^6^ of live singlet cells. TCRβ^+^ thymocytes subpopulations are gated as follow: DN (CD4^-^CD8^-^), DP (CD4^+^CD8^+^), CD4 SP (CD4^+^CD8^-^), CD8 SP (CD4^-^CD8^+^). **(G)** Levels of the indicated mRNAs isolated from WT, *Axl^-/-^Mertk^-/-^*, *Axl^-/-^* and *Mertk^-/-^* thymuses, quantified by RT-qPCR relative to WT. **(H**-**I)** Expression of TCR activation markers, CD5 **(H)** and CD69 **(I)**, as indicators of self-reactivity, measured by flow cytometry. Graphs show normalized mean fluorescence intensity (MFI) of TCRβ^+^ thymocyte subpopulations gated on live singlet cells. (The MFI of one randomly selected WT mouse from each experiment was set to 1, and the MFI for each of the remaining biological samples in the experiment was calculated relative to this WT value.) **(J)** Flow cytometry of T regulatory cells (TCRβ^+^CD4^+^Foxp3^+^) from 1 mo WT and *Axl^-/-^Mertk^-/-^* thymuses. Data are represented as number of Tregs in 10^6^ of live singlet cells. **D**, Representative images from 2-3 mice per genotype. Scale bar, 500μm. **(A–C, E–J)** Graphs are means ± SEM: **E**, **F**, **H**, **I**, **J** are means from at least 3 independent experiments. Each data point represents one separate mouse. The number of mice per genotype are: **A**–**C**, n=10-14; **E**, **J**, n=7; **F**, n=8; **G**, n=5-6; **H**, **I**, n=5-7. *p < 0.05, **p < 0.01, ***p < 0.001 and ****p <0.0001. **A**-**C**, **E**, **F**, **H**-**I**, Unpaired t-test; **G**, One-way ANOVA followed by Tukey’s multiple comparison test.

Supplementary Figure 2 Maintenance of R1 population deficits in the *Axl^-/-^Mertk^-/-^* thymus and spleen at 3 months of age. **(A, B)** Single cell suspensions from 3 mo WT and *Axl^-/-^Mertk^-/-^* thymuses **(A)** and spleen **(B)** were stained and analyzed by flow cytometry. Representative cytometry plots (left in both panels) and quantification (right in both panels) of F4/80^+^CD11b^lo^ (R1) cells. Frequencies calculated as the percent of live singlet cells. **(C)** (Left) Representative cytometry plots of VCAM1, TIM-4 and CD163 expression on F4/80^+^CD11b^lo^ (R1) cells. (Right) Frequencies of TIM-4^+^ and TIM-4^-^, VCAM1^+^ and VCAM1^-^, and CD163^+^ and CD163^-^ cells, calculated as the percent of R1, in 3 mo WT and *Axl^-/-^Mertk^-/-^* spleen. Graphs are mean ± SEM from at least 3 independent experiments. Each data point represents a separate mouse: **(A)** n=8-9 mice per genotype, **(B, C)** n=9-11 mice per genotype. *p < 0.05 and ****p <0.0001. One-way ANOVA followed by Tukey’s multiple comparison test.

Supplementary Figure 3 Depletion of Axl^+^Mer^+^ macrophages from the bone marrow but maintenance of Axl^-^Mer^+^ microglia in the brain of *Axl^-/-^Mertk^-/-^* mice. **(A, B)** Single cell suspensions from 1mo WT and *Axl^-/-^Mertk^-/-^* bone marrow were stained and analyzed by flow cytometry. Representative cytometry plots **(A)** and quantification **(B)** of F4/80^+^CD11b^lo^ (R1) and F4/80^+^CD11b^hi^ (R2) cells. Frequencies calculated as the percent of live singlet cells. **(C)** Representative histograms indicating macrophage marker expression (VCAM1, TIM-4, CD163, Axl and Mer) in R1 (green) and R2 (grey) populations from WT bone marrow. VCAM1, TIM-4, CD163, Axl and Mer are exclusively expressed in R1. Single cell suspension from 1-mo WT and *Axl^-/-^Mertk^-/-^* brains were stained and analyzed by flow cytometry. Representative cytometry plots **(D)** and quantification **(E)** of microglia gated on CD45^+^CD11b^+^. Frequencies calculated as the percent of live singlet cells. **(B–E)** Graphs are mean ± SEM from 2 **(B)** and 3 **(E)** independent experiments. Each data point represents one separate mouse: **B**, n=5 mice per genotype; **E**, n=7 mice per genotype. *p < 0.05. Unpaired *t*-test.

Supplementary Table 1 Antibodies.
