# Supplementary figures and images for: The TAM receptor tyrosine kinases Axl and Mer drive the maintenance of highly phagocytic macrophages
Source: Front Immunol. 2022 Jul 29;13:960401. doi: 10.3389/fimmu.2022.960401 (PMC9373726; doi:10.3389/fimmu.2022.960401)

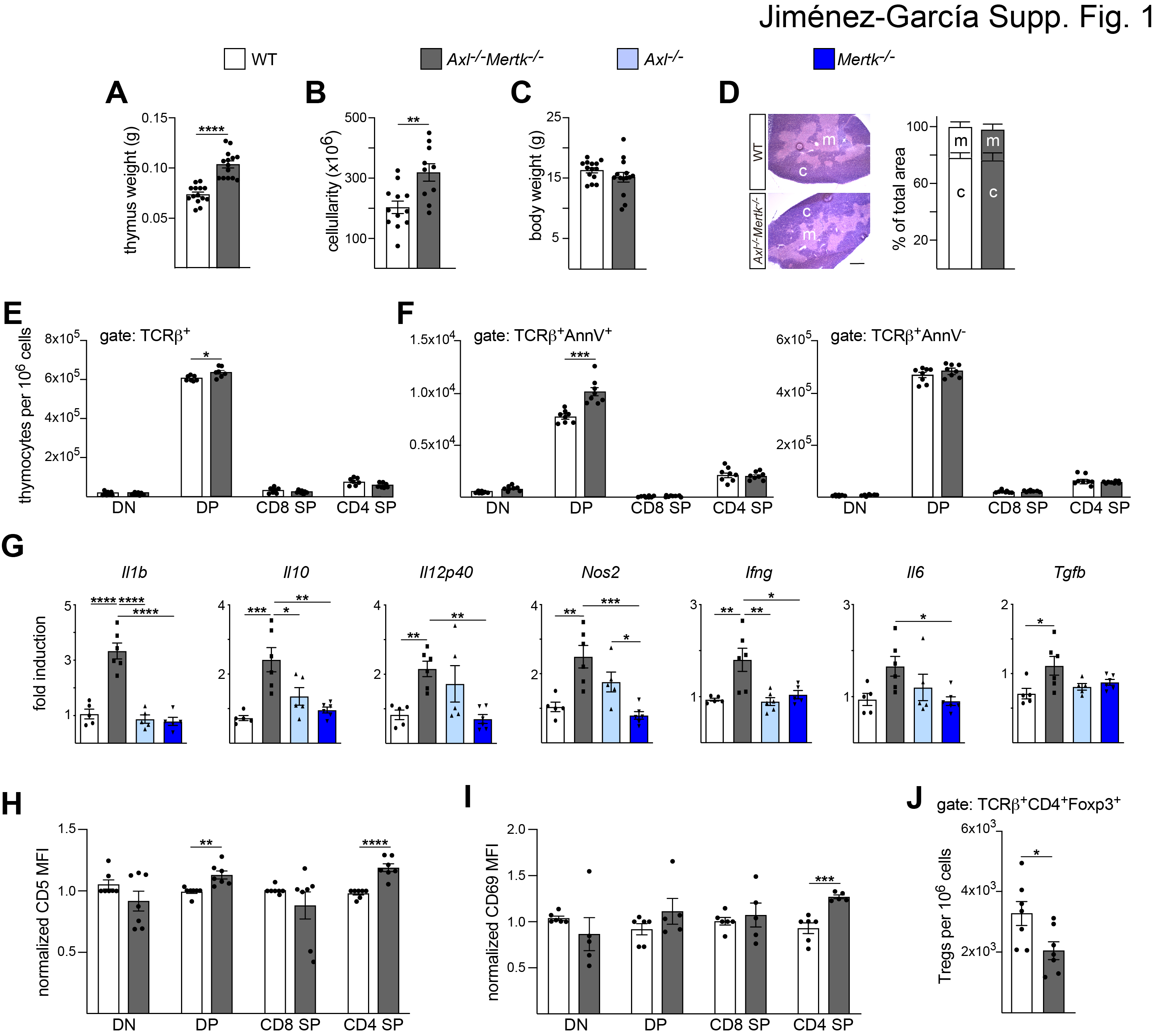

Supplement: Supplementary Figure 1 — Cellular, inflammatory, and autoimmune perturbations in the Axl-/-Mertk-/- thymus. Increased (A) thymus weight and (B) cellularity in Axl-/-Mertk-/- compared to WT mice at 1 mo. No change in (C) body weight is evident between genotypes. (D) Representative images of 1 mo thymic sections stained with H&E (left), and quantification (right) of the ratio cortex (c) and medulla (m) represented as the percent of total area. (E) Quantification of TCRβ+ thymocyte subpopulations gated on live singlet cells from 1 mo WT and Axl-/-Mertk-/ - mice, analyzed by flow cytometry. (F) Thymocytes assayed for apoptosis by flow cytometry. Propidium iodide (PI) positive cells were discarded (dead cells). Only viable (PI-) singlet TCRβ+ thymocytes were segregated and analyzed based in their AnnexinV (AnnV) staining: TCRβ+ AnnV+ thymocytes (viable, early apoptotic) and TCRβ+ AnnV- thymocytes (viable, non-apoptotic). (E, F) Data are represented as number of TCRβ+ thymocytes in 106 of live singlet cells. TCRβ+ thymocytes subpopulations are gated as follow: DN (CD4-CD8-), DP (CD4+CD8+), CD4 SP (CD4+CD8-), CD8 SP (CD4-CD8+). (G) Levels of the indicated mRNAs isolated from WT, Axl-/-Mertk-/- , Axl-/- and Mertk-/- thymuses, quantified by RT-qPCR relative to WT. (H-I) Expression of TCR activation markers, CD5 (H) and CD69 (I), as indicators of self-reactivity, measured by flow cytometry. Graphs show normalized mean fluorescence intensity (MFI) of TCRβ+ thymocyte subpopulations gated on live singlet cells. (The MFI of one randomly selected WT mouse from each experiment was set to 1, and the MFI for each of the remaining biological samples in the experiment was calculated relative to this WT value.) (J) Flow cytometry of T regulatory cells (TCRβ+CD4+Foxp3+) from 1 mo WT and Axl-/-Mertk-/- thymuses. Data are represented as number of Tregs in 106 of live singlet cells. D, Representative images from 2-3 mice per genotype. Scale bar, 500μm. (A–C, E–J) Graphs are means ± SEM: E, F, H, I, J are means from [file Image_1.jpg]

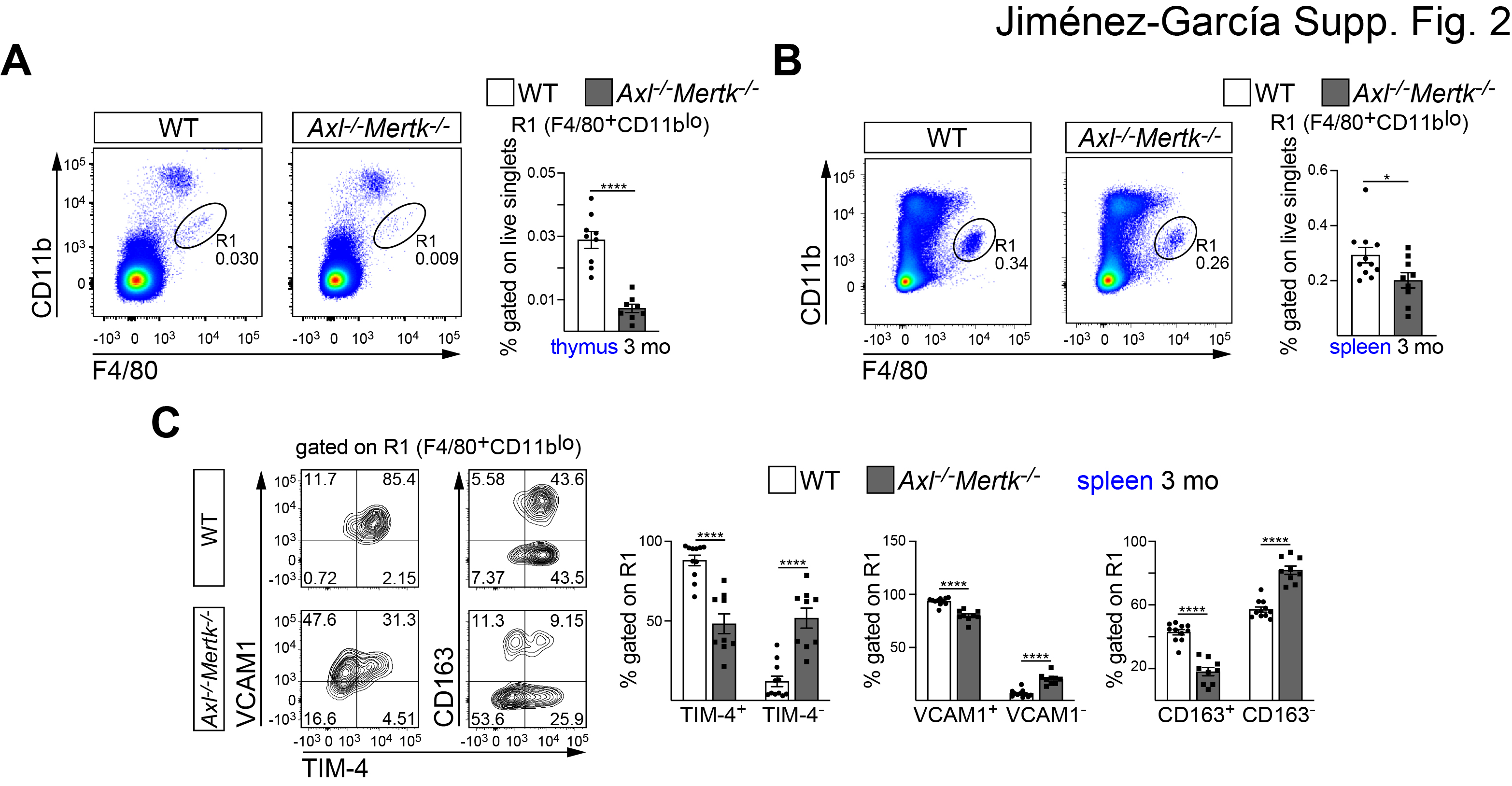

Supplement: Supplementary Figure 2 — Maintenance of R1 population deficits in the Axl-/-Mertk-/- thymus and spleen at 3 months of age. (A, B) Single cell suspensions from 3 mo WT and Axl-/-Mertk-/- thymuses (A) and spleen (B) were stained and analyzed by flow cytometry. Representative cytometry plots (left in both panels) and quantification (right in both panels) of F4/80+CD11blo (R1) cells. Frequencies calculated as the percent of live singlet cells. (C) (Left) Representative cytometry plots of VCAM1, TIM-4 and CD163 expression on F4/80+CD11blo (R1) cells. (Right) Frequencies of TIM-4+ and TIM-4-, VCAM1+ and VCAM1-, and CD163+ and CD163- cells, calculated as the percent of R1, in 3 mo WT and Axl-/-Mertk-/- spleen. Graphs are mean ± SEM from at least 3 independent experiments. Each data point represents a separate mouse: (A) n=8-9 mice per genotype, (B, C) n=9-11 mice per genotype. *p < 0.05 and ****p <0.0001. One-way ANOVA followed by Tukey’s multiple comparison test. [file Image_2.jpg]

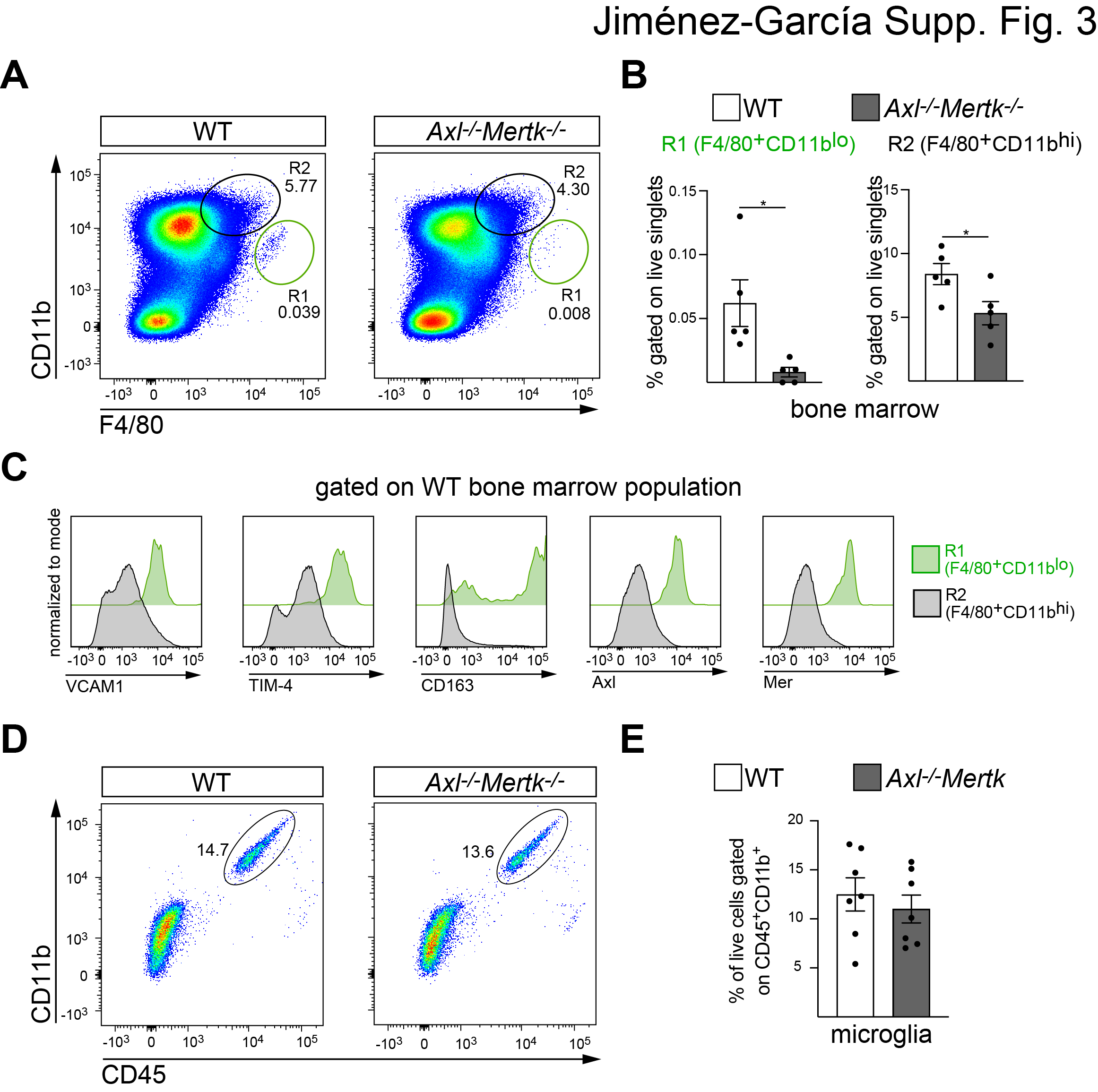

Supplement: Supplementary Figure 3 — Depletion of Axl+Mer+ macrophages from the bone marrow but maintenance of Axl-Mer+ microglia in the brain of Axl-/-Mertk-/- mice. (A, B) Single cell suspensions from 1mo WT and Axl-/-Mertk-/- bone marrow were stained and analyzed by flow cytometry. Representative cytometry plots (A) and quantification (B) of F4/80+CD11blo (R1) and F4/80+CD11bhi (R2) cells. Frequencies calculated as the percent of live singlet cells. (C) Representative histograms indicating macrophage marker expression (VCAM1, TIM-4, CD163, Axl and Mer) in R1 (green) and R2 (grey) populations from WT bone marrow. VCAM1, TIM-4, CD163, Axl and Mer are exclusively expressed in R1. Single cell suspension from 1-mo WT and Axl-/-Mertk-/- brains were stained and analyzed by flow cytometry. Representative cytometry plots (D) and quantification (E) of microglia gated on CD45+CD11b+. Frequencies calculated as the percent of live singlet cells. (B–E) Graphs are mean ± SEM from 2 (B) and 3 (E) independent experiments. Each data point represents one separate mouse: B, n=5 mice per genotype; E, n=7 mice per genotype. *p < 0.05. Unpaired t-test. [file Image_3.jpg]
